# Exacerbation of Intracranial and Gastrointestinal Bleeding in Patients Above 50 Years of Age Co-treated With Antidepressants and Anticoagulants/Platelet Inhibitors at a Lebanese University Hospital

**DOI:** 10.7759/cureus.84033

**Published:** 2025-05-13

**Authors:** Khalil Richa, Elie El Batrouni, Jeannette Sarkis, Rita Abdo, Sandra Aad, Issam Kassab, Mirna N Chahine

**Affiliations:** 1 Department of Emergency Medicine, Lebanese University Faculty of Medicine, Beirut, LBN; 2 Department of Urology, Lebanese University Faculty of Medicine, Beirut, LBN; 3 Department of Internal Medicine, Lebanese University Faculty of Medicine, Beirut, LBN; 4 Department of Neurology, Lebanese University Faculty of Medicine, Beirut, LBN; 5 Department of Pediatrics, Lebanese University Faculty of Medicine, Beirut, LBN; 6 Department of Pharmacology, Lebanese University Faculty of Pharmacy, Beirut, LBN; 7 Department of Basic Sciences, Lebanese University Faculty of Medicine, Beirut, LBN

**Keywords:** anticoagulants, antidepressants, antiplatelets, drug-drug interaction, gastrointestinal bleeding, intracranial bleeding, platelet inhibitors, serotonin

## Abstract

Background and objective: Depression is prevalent in the elderly, to whom the administration of anticoagulants (AC) and antiplatelets (antiaggregants (AG)) is frequent. Antidepressants (AD) work on increasing serotonin levels in the central nervous system (CNS) and are thought to decrease serotonin levels in platelets, an important factor in homeostasis. The effect of the co-administration of AD and AC/AG on increased bleeding risk is controversial; hence, our objective is to determine the effect of AD and AC/AG co-administration on the exacerbation and severity of bleeding in the elderly.

Subjects and methods: A monocentric retrospective study was conducted at the Lebanese Hospital Geitaoui by reviewing charts of 135 patients with gastrointestinal bleeding (GIB) and intracranial bleeding (ICB) hospitalized between 2015 and 2020. Proportions of patients treated with AD and AG, AD and AC, AD alone, or untreated with AD were determined. The studied parameters were demographic variants, medical and surgical histories, medication profiles, hemostasis tests, computed tomography (CT) scans, magnetic resonance imaging (MRI), endoscopies, and characteristics of the bleeding classified according to the Bleeding Severity Measurement Score (BSMS).

Results: No positive correlation was found between AD and AC/AG co-administration and bleeding. Factors such as gender, age, body mass index (BMI), alcoholism, smoking, hypertension, diabetes, history of malignancy, dyslipidemia, chronic kidney disease (CKD), and coronary artery disease (CAD) were not statistically associated with intracranial hemorrhage (ICH) or GIB. Prothrombin time (PT) and international normalized ratio (INR) were significantly (p < 0.05) associated with bleeding severity. Multivariate analysis showed that bleeding severity increases with the clinical diagnosis (p < 0.001) and in patients with heart failure (p = 0.023).

Conclusion: Our study could not prove the potential drug-drug interaction (DDI) between AC/AG and AD. With the increased prevalence of mental disorders and prescribed antidepressants, proving the potential drug-drug interaction is of major importance. More research with diverse approaches on a larger scale is required in this regard.

## Introduction

Serotonin (5-hydroxytryptamine (5-HT)) is a neurotransmitter known to regulate mood, sleep, and memory, among other functions, including hemostasis [1]. In the case of depression and other mental disorders, 5-HT and other neurotransmitters, such as norepinephrine, are deficient in particular sites of the brain [1,2]. Serotonin reuptake inhibitors (SRIs) (selective serotonin reuptake inhibitors (SSRIs) (e.g., fluoxetine) and serotonin and norepinephrine reuptake inhibitors (SNRIs) (e.g., venlafaxine)) act on counterbalancing monoamine deficiency by preventing their reabsorption by nerve terminals at the presynaptic membrane, allowing to boost their synaptic levels to accumulate at the postsynaptic nerve terminal membrane [2].

Concerning its hemostatic role, 5-HT is involved in platelet aggregation. In detail, the serotonin stored physiologically in platelet granules is released into the blood upon the initiation of platelet aggregation to activate 5-HT 2A receptors on the platelet membrane, which enhances the aggregation process [3]. SSRIs and SNRIs can inhibit the 5-HT reuptake in platelets, inhibiting its storage in dense granules. So, the SRI binds to the serotonin transporter expressed on the platelet cell membrane, decreases the 5-HT uptake by platelets, and leads to an inhibition of platelet aggregation mechanisms. This disruption of platelet activation increases bleeding risks [3,4].

Various studies and reviews described an increased bleeding risk or severity in patients using SRIs, particularly gastrointestinal bleeding (GIB) and intracranial hemorrhage (ICH) [5-8]. On the other hand, bleeding risk, particularly GIB and ICH, is known to be present in patients using anticoagulants (AC) and platelet inhibitors (antiaggregants (AG)) [9-12].

In this context, it is noteworthy that the use of SRIs is prevalent in older people for whom the administration of AC and AG is frequent. This is attributed to the fact that mental disorders, on the one hand, are frequent in the elderly due to many factors, including neurological and cardiovascular diseases, and that, on the other hand, necessitates long-term therapy with AC and/or AG [13-17]. Consequently, various studies were performed to indicate that SRIs co-administered with AC or AG enhance the risk of bleeding. However, the results are controversial. For instance, the risk of ICH under SSRIs increases when used concomitantly with oral anticoagulants [7], and the bleeding risk, particularly GIB, increases with the combination of duloxetine (SNRI) with nonsteroidal anti-inflammatory drugs (NSAIDs), including aspirin [8]. Conversely, a study done in 2011 tried but could not prove a significantly increased bleeding risk in patients exposed at the same time to SSRIs and AG as compared to AG exposure alone [18]. Furthermore, a meta-analysis done in 2014 suggested a significant risk of upper gastrointestinal bleeding when SSRIs are administered with AG and NSAIDs. However, this meta-analysis suggested more extensive studies to confirm this interaction [6]. In addition, a systematic review and meta-analysis published in 2022 indicated that using SRIs among patients treated with AC or AG is associated with higher bleeding risk but recommended further research with higher evidence strength to prove the interaction [19].

Adding to this controversy is the need for more similar studies in Lebanon. This study was designed with the primary objective of assessing the incidence and severity of intracranial bleeding (ICB) and gastrointestinal bleeding in patients above 50 years of age who are co-treated with serotonin reuptake inhibitors (SRIs) and anticoagulants or platelet inhibitors in Lebanon.

The secondary objectives were to assess if these events are dependent, first, on treatment regimens, such as the SRI co-administration with an antiaggregant (AG) or an anticoagulant (AC), and second, on the types, administered doses, and duration of therapy of AD, AG, AC, and other home medications. The data analysis tried to link the bleeding events and their severities to the social history (smoking and alcoholism), demographic characteristics (gender, age, and marital status), and past medical history of the cases (presence of one or more comorbidities).

## Materials and methods

Ethical considerations

Our study was conducted after the approval of the Faculty of Medical Sciences thesis committee at Lebanese University. On December 21, 2020, the Institutional Review Board (IRB) of Lebanese Hospital Geitaoui (code: 2020-IRB-039) granted ethical approval to access the patient's medical files.

Our research caused no harm to the patients since the data was mainly collected retrospectively from patients' archived medical files. Moreover, study codes replaced the patients' names, and all information gathered was kept confidential. Missing data was collected by phone, calling the patients or their family members. Phone calls were made using a script written in Arabic and English and approved by the hospital's IRB.

Study design and population

This was a retrospective, monocentric cohort study conducted at the Lebanese Hospital Geitaoui. All subjects included in the study were adults aged more than 50 years old, presenting with intracranial or gastrointestinal bleeding. We excluded subjects presenting with bleeding events other than the intracranial or gastrointestinal bleed, patients who underwent any recent intracranial or gastrointestinal surgical procedure, patients having any bleeding abnormalities (thrombocytopenia, coagulopathies, etc.), patients having head trauma events, and patients treated by AC and AG simultaneously.

Then, based on the information retrieved from their previous medical history section in their medical file, we determined the proportions of patients treated with AD and AG, treated with AD and AC, treated with AD only, and untreated with AD regardless of other medications.

Data collection

Data was collected after IRB approval between January 1 and the end of July 2021.

Patients' files were collected from a single center, the Lebanese Hospital Geitaoui. Data included the bleeding cases over a six-year interval, from January 2015 to December 2020.

For each patient meeting the inclusion criteria, we reviewed patients' medical files according to the patient's presentation (intracranial or gastrointestinal bleeding) and considered the following parameters and examinations: laboratory tests: prothrombin time (PT), international normalized ratio (INR), and partial thromboplastin time (PTT); imaging: brain computed tomography (CT) scan and magnetic resonance imaging (MRI); endoscopies including gastroscopy and colonoscopy; and finally, the list of home medications.

In addition, bleeding severity was assessed using the Bleeding Severity Measurement Score (BSMS), developed in 2012 by Webert et al., which classifies patients into five total subcategories of bleeding severity [20]. The BSMS divides bleeding first into two main categories: non-clinically significant bleeding and clinically significant bleeding. Clinically insignificant bleeding does not affect the patient or the treatment delivered. It is divided into trace bleeding, which can only be discovered by laboratory tests, and moderate bleeding, which can be recognized clinically. Clinically significant bleeding, on the other hand, is subdivided into severe bleeding, serious bleeding producing substantial morbidity, and fatal bleeding [20].

We also collected and studied the complete list of home medications for each patient included in this study. Drug-drug interactions (DDIs) were studied in the study population based on the "Thesaurus of drug interactions" from the ANSM (L'Agence nationale de sécurité du médicament et des produits de santé) [21].

The medical files from which information was retrieved needed more requested parameters, such as the patient's weight, height, and duration of treatment. Solving this obstacle was done by calling the patient by phone.

Data analysis

All statistical analysis was performed using IBM SPSS version 25 (IBM Corp., Armonk, NY). A descriptive analysis was conducted, and the variables were presented according to their type. Categorical variables were presented as frequencies and proportions. Continuous variables were presented as frequencies, means, medians, and standard deviations. A statistical analysis compared the severity of bleeding events in the different groups. The tests used are the Chi-square test and Fisher's exact test. A correlation was considered statistically significant when the P value was less than 0.05 (alpha error = 5%).

Based on Cochran's formula, using Z = 1.96 for a 95% confidence level and e = 0.05 as the desired margin of error, and considering the rarity of concurrent treatment with SRIs and anticoagulants or platelet inhibitors in patients over 50 years of age, a sample size of 135 patients is deemed reasonably representative for the purposes of this study.

## Results

Demographic characteristics

A total of 135 patients were included in the study. Patients were distributed between 83 males (61.5%) and 52 females (38.5%) with an average age of 75.92 ± 11.24 years and a median age of 78. Most patients were not obese, with a mean body mass index (BMI) of 25.09 ± 4.20 kg/m^2^. Among the 135 patients, 103 (76.3%) were married, and 30 (22.2%) were singles. Regarding habits, 39 (28.9%) were active smokers, and 24 (17.8%) were alcoholics.

Comorbid diseases

Out of 135 patients, 94 (69.6%) had hypertension, 44 (32.6%) had diabetes, 16 (11.9%) had a personal history of malignancy, 53 (39.3%) had dyslipidemia, 15 (11.1%) had chronic kidney disease (CKD), and eight (5.9%) had hypothyroidism. Regarding cardiovascular diseases or events requiring anticoagulant (AC)/antiaggregant (AG) therapy, 39 (28.9%) patients had coronary artery disease (CAD), 16 (11.9%) had percutaneous transluminal coronary angioplasty (PTCA), 16 (11.9%) had atrial fibrillation (AFIB), 12 (8.9%) underwent coronary artery bypass graft (CABG) surgery, 15 (11.1%) had heart failure, four (3%) underwent mechanical valve replacement, four (3%) had a history of ischemic stroke, and 17 (12.6%) were taking AC or AG for an unspecified cause. Regarding disorders requiring AD, 3% of all included patients had depression, and AD treated 8.9% for an unspecified reason. Concerning old surgical histories, out of 135 patients, 19 (14.1%) underwent abdominal surgery, and three (2.2%) underwent brain surgery.

Medications

After reviewing the medications of interest in the lists of home medications of the 135 included patients, we found that 61 patients are treated by AG, with a prevalence of 45.2%. AG was mainly used as monotherapy. The used AGs were mainly acetylsalicylic acid 100 mg (n = 38, 62.3%) and clopidogrel 75 mg (n = 14, 23%). Regarding AC, 18 patients used AC as home medication, with a prevalence of 13.3% in our study population. The ACs were mostly acenocoumarol (n = 15, 83.3%) and dabigatran etexilate (n = 3, 16.7%). The prevalence of patients treated with AD as home medication was 8.9% (n = 12 of 135). The ADs mostly used were escitalopram (n = 6, 50%) and sertraline (n = 4, 33.3%) (Table 1).

**Table 1:** Treatment with antiaggregants, anticoagulants, or antidepressants in the study population (N = 135) AG: antiaggregant, AC: anticoagulant, AD: antidepressant, SSRI: selective serotonin reuptake inhibitor, SNRI: serotonin and norepinephrine reuptake inhibitor

Variable		Frequency	Percentage
Use of platelet inhibitor (AG) as home medication	No	74	54.8
	Yes	61	45.2
If yes, monotherapy or dual AG therapy	Monotherapy	50	82.0
	Dual antiplatelet therapy	11	18.0
If yes, the first or unique AG is:	Acetylsalicylic acid 81 mg od	7	11.5
	Acetylsalicylic acid 100 mg od	38	62.3
	Clopidogrel 75 mg od	14	23.0
	Ticagrelor 90 mg bid	1	1.6
	Aspirin 100 mg 1 tablet po q 10 days	1	1.6
Duration	Between 1 and 3 months	2	3.3
	Between 3 and 6 months	3	4.9
	More than six months	56	91.8
If dual AG therapy, the second AG drug is:	Clopidogrel 75 mg od	8	72.7
	Ticagrelor 90 mg bid	3	27.3
Duration	Between 3 and 6 months	2	18.2
	More than six months	9	81.8
Use of AC as home medication	No	117	86.7
	Yes	18	13.3
If yes, which one	Acenocoumarol (Sintrom)	15	83.3
	Dabigatran etexilate	3	16.7
Duration	Between 1 and 3 months	2	11.1
	Between 3 and 6 months	1	5.6
	More than six months	15	83.3
Use of AD (SSRI/SNRI) as home medication	No	123	91.1
	Yes	12	8.9
If yes, the used AD is:	Escitalopram (Cipralex, Lexapro)	6	50.0
	Sertraline (Zoloft)	4	33.3
	Paroxetine (Paxil, Pexeva, Seroxat)	1	8.3
	Fluoxetine (Prozac, Sarafem)	1	8.3
Duration	More than six months	12	100.0

**Figure 1:**
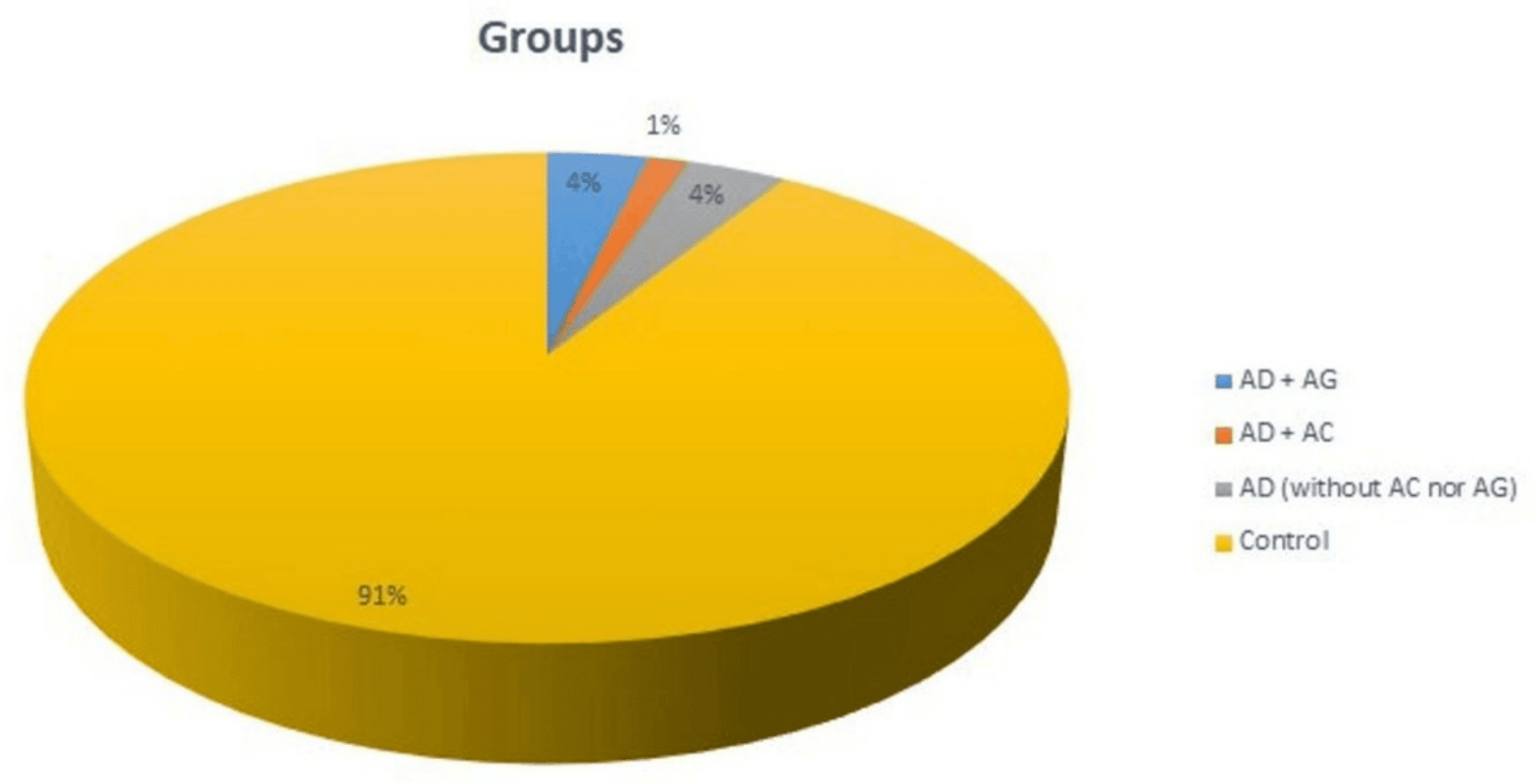
Distribution of patients who received a combination of AD and AG, a combination of AD and AC, and AD only, and control AG: antiaggregants, AC: anticoagulants, AD: antidepressants

Out of 135 patients having bleeding, five (3.7%) were treated with the combination AD/AG, two (1.5%) were treated with the combination AD/AC, five (3.7%) were treated with only AD (without AC/AG), and 123 (91.1%) were treated with only AC or AG or other treatments (Figure 1).

The prevalence of patients treated with proton pump inhibitor (PPI) as home medication was 31.1% (n = 42 of 135). The most commonly received PPIs were omeprazole (n = 14, 50%), esomeprazole (n = 12, 29.3%), and rabeprazole sodium (n = 9, 22%). Most patients were treated with PPIs for over six months (n = 37, 90.2%). The analysis of known drug-drug interactions (DDIs) showed that the average number of significant known DDIs was 0.32 ± 0.74, with a minimum of 0 significant DDIs and a maximum of four significant DDIs.

Laboratory findings

In the study population, the PT values ranged from 10.40 to 98.50, with a median PT of 12.5 (interquartile range (IQR): 11.50-14.50). The PTT ranged between 11.50 and 70, with a median of 26 (IQR: 24.00-29.00). The median INR was 1.1 (IQR: 1.02-1.31), with a minimum of 0.9 and a maximum of 11.5.

Bleeding-related characteristics

Type of Bleeding

Out of 135 bleeding cases, 63 (46.6%) had gastrointestinal bleeding, and 72 (53.3%) had intracranial bleeding. In detail, bleeding was shown in the upper GI in 23 (17%) patients, lower GI in 40 (29.6%) patients, and intracranial in 72 (53.3%) patients.

Examinations to Detect Bleeding

Bleeding was detected through brain CT (n = 64, 47.4%), brain MRI (n = 15, 11.1%), gastroscopy (n = 18, 13.3%), colonoscopy (n = 22, 16.3%), clinical diagnosis (n = 54, 40%), and other examinations (n = 6, 4.3%) (abdominopelvic CT scan, ultrasound, and video capsule).

Intracranial Bleeding

Among the 72 patients who presented with intracranial bleeding, 41 (56.9%) had subdural hemorrhage, 19 (26.4%) had intracerebral hemorrhage, and 13 (18.1%) had subarachnoid hemorrhage. Intracranial bleeding was acute in 49 (68.1%), subacute in 15 (20.8%), and chronic in eight (11.1%) patients. Forty-three (59.7%) patients with intracranial bleeding had a history of recent head trauma.

GI Bleed Diagnosis

Among the 23 patients who presented with upper GI bleed, 11 (47.8%) had gastric ulcers, and eight (34.8%) had undetermined causes for upper GI bleed. Among the 40 patients who presented with lower GI bleed, 11 (27.5%) had hemorrhoids or fissures, five (12.5%) had angiodysplasia, and 13 (32.5%) had undetermined causes for lower GI bleed. Among the 63 patients who presented with GI bleed (upper GI and lower GI), 54 (84.4%) had acute bleeding, and nine (14.1%) had chronic bleeding (Table 2).

**Table 2:** GI bleed-related characteristics GI: gastrointestinal, FOBT: fecal occult blood test, FIT: fecal immunochemical test

Variable		Frequency	Percentage
Upper GI bleed diagnosis	Duodenal injury by biliary stent	1	4.3
	Duodenal vascular malformation	1	4.3
	Esophageal varices	1	4.3
	Gastric ulcer	11	47.8
	Tumor (esophageal, gastric, and duodenal)	1	4.3
	Undetermined	8	34.8
Lower GI bleed diagnosis	Angiodysplasia	5	12.5
	Colorectal tumor	2	5.0
	Diverticular disease	4	10.0
	Hemorrhoids/fissure	11	27.5
	Inflammatory bowel disease	3	7.5
	Polyps	2	5.0
	Undetermined	13	32.5
GI bleeding	Acute/overt bleed	54	84.4
	Chronic/occult bleed (FOBT/FIT)	9	14.1

Severity Description of GI Bleed

Bleeding severity was assessed using the Bleeding Severity Measurement Score (BSMS). The severity of bleeding was found to be mainly attributed to the presence of pain (53.2%) and the need for blood transfusion (61.9%). A descriptive analysis of BSMS items is presented in Table 3.

**Table 3:** Bleeding Severity Measurement Scale items BP: blood pressure, MAP: mean arterial pressure

Variable		Frequency	Percentage
Presence of pain	No	29	46.8
	Yes	33	53.2
Needed transfusion	No	24	38.1
	Yes	39	61.9
Needed surgery	No	59	93.7
	Yes	4	6.3
Needed non-surgical invasive procedures (endoscopy or interventional radiology)	No	49	77.8
	Yes	14	22.2
Administration of medications for bleeding management (e.g., octreotide)	No	49	77.8
	Yes	14	22.2
Critical unit admission	No	44	69.8
	Yes	19	30.2
Tachycardia, defined as an increase in resting heart rate by at least 20 bpm or >100	No	50	79.4
	Yes	13	20.6
Hypotension, defined as a decrease in systolic or diastolic blood pressure BP by at least 20 mmHg or BP < 90/60 mmHg or MAP <65	No	45	71.4
	Yes	18	28.6
Vision loss	No	63	100.0
Need for norepinephrine and/or dobutamine	No	55	87.3
	Yes	8	12.7
Presence of any other secondary complication such as hepatic/renal injury/failure, secondary heart ischemia, and brain hypoxia	No	52	82.5
	Yes	11	17.5
Did the bleeding lead to the patient's death?	No	120	88.9
	Yes	15	11.1

Bleeding Severity

**Figure 2:**
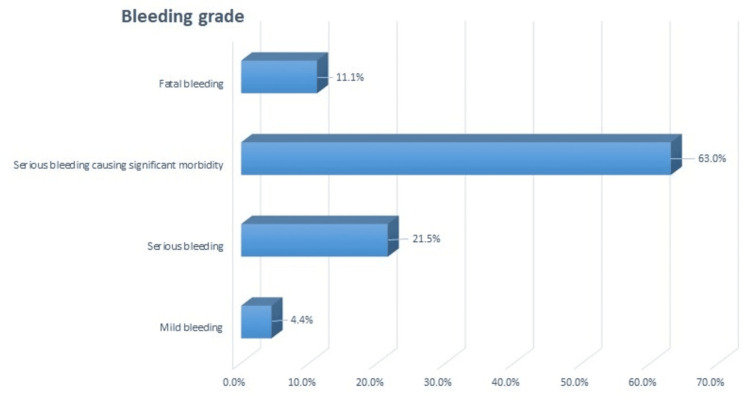
Bleeding grade in the study population

Bleeding severity was categorized based on the BSMS into four categories, and the results showed that 15 (11.1%) patients had fatal bleeding, 85 (63%) had serious bleeding causing significant morbidity, 29 (21.5%) had serious bleeding, and six (4.4%) had mild bleeding (Figure 2). Note that our study population had no patients with trace bleeding severity because all patients had bleeding recognized clinically since they were hospitalized with hemorrhagic events.

Bleeding and Medications

Bleeding severity was not statistically associated with receiving AG (p = 0.313), AC (p = 0.439), AD (p = 0.084), or combinations (p > 0.05) (Table 4).

**Table 4:** Bleeding grade in function of antiaggregant/anticoagulant/antidepressant use AG: antiaggregant, SSRI: selective serotonin reuptake inhibitor, SNRI: serotonin and norepinephrine reuptake inhibitor, AD: antidepressant, AC: anticoagulant

Variable		Bleeding grade				Chi-square	P value
		Mild bleeding	Serious bleeding	Serious bleeding causing significant morbidity	Fatal bleeding		
Use of platelet inhibitors (AG) as home medication	No (n = 74)	4 (5.4%)	13 (17.6%)	48 (64.9%)	9 (12.2%)	3.563	0.313
	Yes (n = 61)	2 (3.3%)	16 (26.2%)	37 (60.7%)	6 (9.8%)		
Use of platelet inhibitors (AG) as home medication	No (n = 117)	6 (5.1%)	23 (19.7%)	76 (65.0%)	12 (10.3%)	2.707	0.439
	Yes (n = 18)	0 (0.0%)	6 (33.3%)	9 (50.0%)	3 (16.7%)		
Use of SSRI/SNRI (AD) as home medication	No (n = 123)	4 (3.3%)	25 (20.3%)	79 (64.2%)	15 (12.2%)	6.656	0.084
	Yes (n = 12)	2 (16.7%)	4 (33.3%)	6 (50.0%)	0 (0.0%)		
Groups	AD + AG (n = 5)	1 (20.0%)	2 (40.0%)	2 (40.0%)	0 (0.0%)	9.023	0.435
	AD + AC (n = 2)	0 (0.0%)	1 (50.0%)	1 (50.0%)	0 (0.0%)		
	AD (without AC and AG) (n = 5)	1 (20.0%)	1 (20.0%)	3 (60.0%)	0 (0.0%)		
	Control (n = 123)	4 (3.3%)	25 (20.3%)	79 (64.2%)	15 (12.2%)		

Among patients who received AG, 16 (26.2%) had serious bleeding, 37 (60.7%) had serious bleeding causing significant morbidity, and six (9.8%) had fatal bleeding. Among patients who received AC, six (33.3%) had serious bleeding, nine (50%) had serious bleeding causing significant morbidity, and three (16.7%) had fatal bleeding. Among patients who received AD, four (33.3%) had serious bleeding, six (50%) had serious bleeding causing significant morbidity, and none had fatal bleeding.

In the control group, 25 (20.3%) patients had serious bleeding, 79 (64.2%) had serious bleeding causing significant morbidity, and 15 (12.2%) had fatal bleeding (Table 4).

Moreover, even when categorizing bleeding severity BSMS into two categories (mild/serious and serious with morbidities/fatal), bleeding severity was not statistically associated with receiving AG (p = 0.389), AC (p = 0.441), AD (p = 0.078), or combinations (p > 0.05).

Factors Associated With Bleeding Severity

Bleeding severity was not associated with gender (p = 0.913), age (p = 0.278), BMI (p = 0.395), marital status (0.393), smoking (p = 0.364), or alcoholism (p = 0.848). In addition, bleeding severity was not associated with hypertension (p = 0.054), diabetes (p = 0.803), history of malignancy (p = 0.600), dyslipidemia (p = 0.994), CKD (P = 0.136), and CAD (p = 0.209).

Bleeding severity was higher in patients diagnosed with heart failure (p = 0.005). Bleeding severity was associated with a history of abdominal surgery (p = 0.039). Bleeding severity was not associated with a history of brain surgery (p = 0.093) or a history of recent head trauma (p = 0.342).

It is worth mentioning that bleeding severity was not associated with the type of bleeding (p = 0.166), but it was associated with having a clinical diagnosis. In other words, patients presenting with an apparent clinical diagnosis of bleeding (hematemesis, hematochezia, or melena) had a more severe bleeding score (p < 0.001).

Regarding medications, bleeding severity was not associated with receiving AG (p = 0.411), AC (p = 0.129), and AD (p = 0.823). Bleeding severity was also not associated with the number of significant DDI (p = 0.170), the number of home medications without AC/AG/AD/PPI/NSAIDS (p = 0.186), or the number of home medications including AC/AG/AD/PPI/NSAIDS (p = 0.119).

Laboratory results, especially PT and INR, were higher in severe bleeding (high BSMS) (p = 0.002 and p = 0.001, respectively).

Multivariate analysis showed that bleeding severity (BSMS) increases with the presence of heart failure in patients (p = 0.023) and with a clear clinical diagnosis (p < 0.001) (Table 5).

**Table 5:** Factors affecting BSMS BSMS: Bleeding Severity Measurement Score [20]

Variable	Unstandardized coefficients		Standardized coefficients	t	P value	95% confidence interval for B	
	B	Standard error	Beta			Lower bound	Upper bound
Clinical diagnosis	1.805	0.198	0.609	9.108	0.000	1.413	2.196
Heart failure	0.711	0.309	0.154	2.301	0.023	0.100	1.322

## Discussion

This retrospective study has investigated the incidence and severity of intracranial and gastrointestinal bleeding in patients above 50 years of age co-treated with SRIs and anticoagulants/platelet inhibitors and the factors associated with bleeding severity.

To this end, 135 patients meeting the inclusion criteria at the Lebanese Hospital Geitaoui were enrolled. Five (3.7%) patients were on AD/AG and two (1.5%) on AD/AC combinations. In addition, most of the cases suffered from one or more medical comorbidities such as hypertension, diabetes, personal history of malignancy, dyslipidemia, CKD, and hypothyroidism.

Trying to find out the effect of drug-drug interaction on bleeding, our study showed no significant association between bleeding severity and receiving the combinations (p > 0.05) despite most studies showing a link between these co-administrations and bleeding, mainly gastrointestinal bleeding [19,22,23]. For instance, the risk of bleeding was found to increase when platelet inhibitors are combined with SSRIs compared to antiplatelets alone [22]. In addition, in a recently published study, Shao et al. found that among atrial fibrillation patients treated with oral anticoagulants, the co-use of an SSRI was associated with the highest bleeding risk when compared to other antidepressants, particularly SNRIs [23]. Moreover, a recent systematic review and meta-analysis by Nochaiwong et al. concluded that co-using SRI with AC or AG increases the risk of bleeding. However, the review findings were inconclusive concerning the risk of intracranial hemorrhage. The risk of bleeding was attributed to many mechanisms, including platelet aggregation inhibition, increasing vagal tone leading to enhanced gastric acidity, and CYP 450 inhibition of SRIs [19].

The difference between results could be due mainly to the fact that our study was conducted on a small sample compared with the other studies, among other limitations.

Bleeding risk factors

We assessed different factors to detect their effect on bleeding incidence and severity. In our study, we did not find a statistically significant association between bleeding events and many factors, such as gender, BMI, smoking, alcohol consumption, and most of the patients' medical comorbidities.

However, our first significant finding is that bleeding occurrence and severity are significantly correlated with the presence of underlying heart failure. This significance is in accordance with the literature showing that heart failure is strongly related to bleeding events, whether GIB or ICH, partially attributed to the concomitant use of AC and/or AG [24,25].

Besides heart failure, the clinical diagnosis of bleeding was statistically correlated with the bleeding events. In fact, the clinical diagnosis of GIB is made mainly through clinical presentations such as hematemesis, hematochezia, and melena, which are primarily cases of acute and active upper or lower GI bleeding that necessitate immediate interventions, consequently increasing the assessed bleeding severity based on the BSMS [20,26,27].

Our study has several limitations that should be considered when interpreting the results. First, the study has a retrospective monocentric design. Unlike a multicentric model, such a design limits the ability to compare findings among centers and the generalization of the results to the general population.

Second, we note the limitations related to the retrospective data collection: missing data and recall bias. Most data was collected from medical files that sometimes lacked important information, such as weight, height, and duration of treatment. Solving this obstacle was done by phone calls, which gave rise to recall bias; patients (mostly elderly patients) or their family members tried to provide approximate answers to our questions, and despite this course of action, we still encountered missing data.

Last, but not least, the number of patients recruited is small statistically, and the number of patients found on ADs was lower than the required number needed to prove a drug-drug interaction. However, we think that the number of bleeding cases on antidepressants was underestimated, as we live in a society that stigmatizes mental diseases. Patients hesitate to report their use of ADs, which is a significant limitation to our results.

## Conclusions

This study showed that bleeding severity in the elderly increases mainly in the presence of heart failure and when the diagnosis is made clinically. We could not find a significant drug-drug interaction when studying the combinations of AD/AG or AD/AC. This could be due mainly to the fact that our study was conducted on a small sample compared with other studies, among other limitations.

However, the safety of these co-prescriptions has not yet been proven or rejected in the literature. Further, larger, multicentric studies are needed to better assess the potential bleeding risk.
